# Congenital Zika Infection and the Risk of Neurodevelopmental, Neurological, and Urinary Track Disorders in Early Childhood. A Systematic Review

**DOI:** 10.3390/v13081671

**Published:** 2021-08-23

**Authors:** Evangelia Antoniou, Eirini Orovou, Paraskevi Eva Andronikidi, Christos Orovas, Nikolaos Rigas, Ermioni Palaska, Angeliki Sarella, Georgios Iatrakis, Chrysa Voyiatzaki

**Affiliations:** 1 Department of Midwifery, Egaleo Park Campus, University of West Attica, Ag. Spyridonos Str., 12243 Egaleo, Greece; eorovou@uniwa.gr (E.O.); nrigas@uniwa.gr (N.R.); epalaska@uniwa.gr (E.P.); asare@uniwa.gr (A.S.); giatrakis@uniwa.gr (G.I.); 2 Evaggelismos General Hospital of Athens, Ypsilandou 45-47, 10576 Athens, Greece; eva_andr@hotmail.com; 3 Department of Products and Systems Design Engineering, University of Western Macedonia, 50100 Kozani, Greece; chorovas@uowm.gr; 4 Department of Biomedical Sciences, Egaleo Park Campus, University of West Attica, Ag. Spyridonos Str., 12243 Egaleo, Greece; cvoyiatz@uniwa.gr

**Keywords:** Zika virus infection, congenital Zika syndrome, congenital Zika infection and neurodevelopment outcomes, congenital Zika infection and neurological outcomes, congenital Zika infection and urinary tract disorders

## Abstract

It was late 2015 when Northeast Brazil noticed a worrying increase in neonates born with microcephaly and other congenital malformations. These abnormalities, characterized by an abnormally small head and often neurological impairment and later termed Congenital Zika Syndrome, describe the severity of neurodevelopmental and nephrological outcomes in early childhood, and the implication of microcephaly at birth. The purpose of the study was to describe the neurodevelopmental outcomes in children exposed to Zika virus during fetal life, with and without microcephaly at birth. The systematic review included research studies about the neurodevelopmental outcomes with and without microcephaly, as well as nephrological outcomes in early childhood. We searched PubMed, Crossref, PsycINFO, Scopus, and Google Scholar publications and selected 19 research articles published from 2018 to 2021. Most studies have linked the severity of microcephaly in childbirth to the neurodevelopmental and urinary outcomes in early childhood. However, most children without microcephaly at birth develop typically, while others may be at risk for language impairment.

## 1. Introduction

The Zika epidemic first caught the global attention in late 2015 when Brazil started reporting an increase in cases of children born with microcephaly potentially linked to the outbreak. Zika is transmitted to humans by the Aedes aegypti mosquito bite, which is the same mosquito that transmits Chikungunya, Dengue, and Yellow Fever. Zika virus can also be transmitted sexually from a person who has Zika virus infection [1]. After this outbreak, the World Health Organization (WHO) declared the Zika virus (ZIKV) infection as a Public Health emergency of global interest [2] and since then investigations have begun into the causal link between zika virus and microcephaly in infants [3,4,5,6,7,8,9,10,11]. Most studies have focused on maternal infection during pregnancy, especially in the first trimester and the beginning or the whole of the second trimester [12]. Microcephaly is characterized by a reduction of more than 2 SDs in the head circumference depending on the sex and age [13] and may be accompanied by seizures, developmental delay, intellectual disability, feeding problems, hearing loss, and vision problems [14].

### 1.1. Congenital Zika Syndrome and Neurodevelopmental Outcomes

The Congenital Zika Syndrome (CZS) is characterized by a distinct pattern of birth defects and disabilities and has been identified with Zika virus infection during pregnancy. Although many of the features seen as part of the CZS can be caused by other pregnancy infections, the characteristics of the CZS are unique and include severe microcephaly (the skull has partially collapsed), decreased brain tissue (including subcortical calcifications), damage to the back of the eye (including macular scarring and focal retinal pigmentary mottling), hypertonia soon after birth, and arthrogryposis [15,16]. Although the initial description of the CZS included the above severe symptoms, it soon became clear that the clinical and neurodevelopmental features of the CZS cover a wide range of severity, ranging from asymptomatic [17] and mild [18] to severely affected children [19].

Congenital microcephaly has been a hallmark of uterine Zika virus infection. However, despite the absence of microcephaly at birth, in some cases with laboratory evidence with Zika virus, there were brain abnormalities associated with CZS [18]. The pathogenesis of postnatal microcephaly from congenital Zika virus infection is still unknown. However, Zika virus can cause an asymptomatic infection at birth, leading subsequently to impaired brain development and the consequences of this phenomenon can become apparent in early childhood [20]. The decrease in head growth might be the consequence of in utero destruction of neural cells, persistent inflammatory response-associated molecules or continued infection of neural cells [18].

### 1.2. Congenital Zika Syndrome and Urinary Track Outcomes

While neurodevelopmental outcomes in children with CZS are more common and obvious, the urinary track outcomes are not so well known and are still under investigation. Mostly insignificant abnormalities from the urinary system have been reported, that seem related to zika virus infection. Several cases of newborns and children with CZS, suffering from neurogenic bladder but not from vesicoureteral reflux, have been announced [21,22]. Particularly, a study update observed to be clearly associated with detrusor over activity [23]. Writers concluded that there is no indication for patients with CZS to be regularly submitted to urodynamic control, and such an assessment should be carried out only when risk factors are present (pyelonephritis, ultrasound abnormalities) [24].

The central nervous system abnormalities are involved in the control of the lower urinary tract system. It seems that the mechanism affecting the urinary system is through the nerve pathways that control the malfunction of the urinary bladder possibly leading to reflux nephropathy. The urinary bladder stores and periodically eliminates urine, a function regulated by a complex neural control system in the brain, spinal cord, and peripheral autonomic ganglia that coordinates the activity of smooth and striated muscles of the bladder and urethral outlet. The CZS appears to affect these areas that manage these functions. Neurogenic bladder occurs when an injury to the nervous system reaches the urinary centers and disrupts the normal function of the bladder and bladder sphincter. This endangers the physiology and hydrodynamic flow of the urinary tract, causing urological dysfunction of varying degrees, which affects kidney function and can cause renal failure if not diagnosed and treated properly. Therefore, the damage caused by CZS seems to be located in areas of the brain that control the lower urinary tract [22].

Understanding the association between the intrauterine Zika virus infection and the development of urinary tract disorders is paramount in trying to fully identify the disorders caused by Zika virus, and therefore appropriate treatment options that can reduce the impact of these complications on affected children.

Therefore, the aim of our systematic review was to describe the effect of microcephaly at birth on the severity of neurodevelopmental, neurological, and urinary track disorders of congenital Zika infection in early childhood.

## 2. Materials and Methods

**Figure 1:**
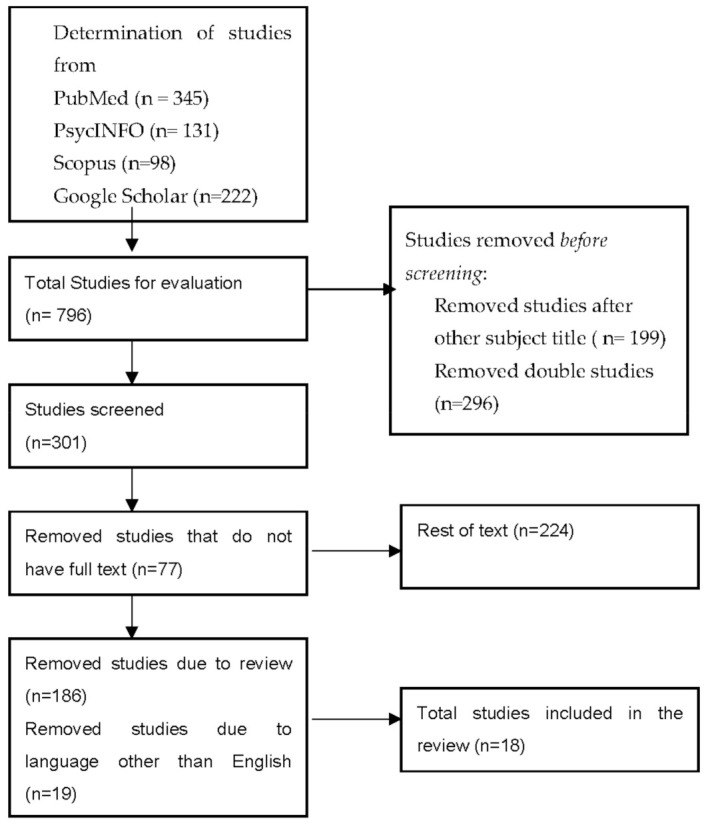
Flow chart-Structure Search Strategy.

The aim of the study was to systematically review the available data from published articles linking causatively the CZS with neurodevelopmental and nephrological outcomes. We conducted a review including research articles on neurodevelopmental outcomes after congenital Zika virus infection. The research was carried out based on PubMed, PsycINFO, Scopus, and Google Scholar publications. The keywords used were: “Congenital Zika virus infection, and children outcomes”, “Congenital Zika virus infection and children disability”, “Congenital Zika virus infection and neurodevelopmental outcomes”, “Congenital Zika virus infection and microcephaly” “Congenital Zika virus infection and nephrological outcomes”. The timeline was set from 2018 to 2021 and out of 796 studies, only 19 were included in the review (Figure 1). The studies were first screened by abstract and title so double studies were removed. The full texts of appropriate studies were examined again and from 224 studies, a total of 186 reviews, systematic reviews and meta-analyses, as well as articles with other languages than English were rejected. The study analyzed the neurodevelopmental and urinary outcomes in children after their intrauterine exposure to Zika virus. All of the participants (mothers and children) had serologic confirmation of Zika virus and met the clinical criteria of suspected exposure to Zika virus during pregnancy and negative laboratory results for toxoplasmosis, cytomegalovirus, syphilis, and rubella.

All of the children underwent neural imaging examinations. For the infant and toddler neurodevelopmental evaluation the appropriate scales were used. For children with urinary track disorders, laboratory tests, renal and bladder ultrasounds, and diagnostic urodynamic studies were used

Regarding the methodological quality of the studies, nine criteria were used to rate them. The first criterion, concerning the representative exposure sample, was met by all articles with the exception of the case report and the clinical one. Regarding the second criterion, no study met that. All of the studies met the third criterion since the exposed children were identified with laboratory tests. In all of the studies, the outcome did not precede the study. The fifth criterion, which was the adaptation for the educational level, exists in eight studies. All of the studies met the sixth criterion. All of the studies met the seventh criterion for the evaluation of Zika virus infection effect in early childhood. Moreover, there was sufficient monitoring time in all of the studies fulfilling the eighth criterion. In the end, all of the studies met non-bias of wear, the ninth criterion. The score of the studies varied between 5 and 9. No research had a score of 9 due to the absence of criterion 2 (Table 1).

## 3. Results

The 19 articles included in this systematic review have been carried out mainly in Brazil. Of the 19 studies, 12 were cohort studies, three cross-sectional, two longitudinal, one clinical study, and one case report (Table 2). Regarding the methodological evaluation of the studies, 10 studies were very good, seven were good, and two were of moderate methodological quality (Table 1). In more detail, in the study of Wheather et al., 2020 [25], all the available data from 121 children support a causal link between CZS (microcephaly) with profound delays in all developmental domains (motor, visual, language, cognitive). According to the Pereira et al. (2020) [26] cohort study, from a total of 75 toddlers, a percentage of 75% was born with microcephaly and a percentage of 25% without microcephaly. Of the 19 children born with a normal head circumference, 15 had a postnatal delay in head growth. The findings of this study showed that severe microcephaly is associated with severe neurological dysfunction (dyskinetic findings, dystonia, epilepsy, persistence of primitive reflexes) and urinary tract infections. The Pecanha et al. (2020) [20] cohort study describes the neurodevelopment of children who were born asymptomatic after their intrauterine exposure to Zika virus but at the age of 2, they showed neurodevelopmental delay in three domains, motor, cognition, and mainly in language. In Mulkey et al. (2020) [27], a cohort study of 70 Colombian exposed infants without CZS, assessment scores deviated from normal scores in multiple areas of development in some children as they became older. The above research links the increased risk of impaired brain development even after an asymptomatic infection at birth, while the cross-sectional study of Ferreira et al. (2018) [28] reinforces this hypothesis by presenting findings of complete disability in most functional categories (mental functions of language, severe motor impairment, eating and sleep disorders) in children with microcephaly.

In the Carvalho et al. (2019) [29] cohort study, the majority of the microcephalic children presented with spastic, gross motor function, cerebral palsy, epilepsy, and extremely low performances on cognitive, language, and motor. Τhe above findings agree with the Bertolli et al. (2020) [30] and Silva et al. (2020) [31] studies, which observed that children with microcephaly were more likely to have severe neurological and neurodevelopmental outcomes compared to children who had only laboratory confirmation of the infection. Moreover, in a Cranston et al. (2020) [32] cohort study, although children with a normal head circumference had fewer neurological abnormalities than children born with microcephaly, those with normal head circumference also had common neurological abnormalities, including abnormal tone, hyperreflexia, congenital neuromotor signs, feeding difficulties, and abnormal brain imaging results.

The head circumference at birth for children with microcephaly has also been associated with severe neurodevelopmental delay, severe neuroimaging findings, dysphagia, spasticity, hyperreflexia, hearing loss and epilepsy, according to the Garcia-Boyano et al. (2020) cohort study [33]. A cohort study of Quilião et al. published in 2020 [34] describes the findings of a cohort of children with CZS in terms of their head circumference and neurological-neurodevelopmental outcomes. The results showed that the majority of children with severe microcephaly developed motor dysfunction and/or epilepsy, compared to children who were normocephalic.

The recent study of Ticona et al. (2021) [35] investigated the relationship between the in-utero Zika virus exposure and the prevalence of developmental alterations in early childhood. It was observed that from a group of women living in a slum community in Brazil, both the asymptomatic and symptomatic Zika virus infection during the antenatal period is associated with mild neurodevelopmental abnormalities in children asymptomatic at birth. Furthermore, the exposed children had a greater risk for cognitive delay and abnormal auditory behavior. In a cohort study of Hcini (2021) [36], data from 129 cases of exposed Zika virus children were collected from a pediatric clinic of French Guiana. In the case of infected children, there was a greater risk of neurological outcomes, even when no brain abnormalities were observed. However, at the age of 3, neurodevelopmental delay was more common in children that were positive at birth compared to those without brain abnormalities. In another cohort study, published in 2020 from Abtibol-Bernardino et al., [37], a series of cases of exposed children, asymptomatic at birth, were described. The majority of children had satisfactory neurodevelopment, with the exception of language which was the most impaired domain. Furthermore, severe neurological disorders (spastic hemiparesis and epilepsy) occurred in a few children, while connected with CZS.

There were five studies in our review that report on the effects of prenatal exposure on Zika virus and the urinary track disorders development. More specifically, in a Costa Monteiro et al. (2018) [22] cohort study, from a sample of 22 children with CZS, all were confirmed with neurogenic bladder and the majority of them with urinary tract infection, multicystic dysplastic kidney disease, kidney stone, and sphincter dyssynergia. Moreover, in a later cohort study of Costa Monteiro et al. (2019) [23], CZS was associated with neurogenic bladder, overactive bladder, urinary tract infection, and abnormal bladder capacity. In addition, a cohort study published by de Medeiros Francilaide Campos (2021) [24], a single urologist performed clinical, urological, urodynamical, laboratory, and ultrasonographical evaluations in 33 children with CSZ. Most of the patients did not have renal abnormalities and presented with a motif of undeveloped and reflex bladder, with non-significant post-void residual urine. More specifically, the patients included in the above studies [22,23], have already been diagnosed with CZS and all the children who were screened for urological problems, already had neurological disorders. The surveys confirmed the neurogenic bladder in 100% of tested children. However, the study of Medeiros Francilaide Campos (2021) [24], disagrees with the methodology of the previous two studies belonging to the same researchers group. The authors argue that the former studies did not include the use of a rectal catheter and this could have contributed to a greater number of false-positive diagnoses of neurogenic bladder. Furthermore, the study concludes that only 33.2% of children had neurogenic bladder and the 59.2% had a pattern of immature and reflex bladder, with no repercussions on the upper urinary tract. In addition, in a subsequent study, it appears that children who have continuous urinary monitoring, have a better prognosis [38]. In the case report by Villamil-Gómez et al. (2019) [39], severe bilateral renal hypoplasia and urinary bladder agenesis have been described in an autopsy of a fetus derived from an early terminated pregnancy (16 weeks) of a mother infected by Zika virus. Complete absence of the urinary bladder, and severe bilateral renal hypoplasia. However, complete urinary bladder agenesis is a very rare abnormality with only a few live cases reported so far [39]. Furthermore, an experimental mechanism of acute kidney injury has been proposed in both newborn and adult mouse models infected by ZIKV according to a Liu et al. (2019) [40] clinical study on mice, where increasing levels of AKI-related biomarkers (e.g., serum creatinine (Scr), kidney injury molecular−1 (Kim-1), and neutrophil gelatinase-associated lipocalin (NGAL)) were identified [40].

## 4. Discussion

The aim of our systematic review was to describe the effect of microcephaly at birth on the severity of neurodevelopmental, neurological, and urinary track disorders of congenital Zika infection in early childhood. We provide strong evidence for a probable link between the presence and the severity of microcephaly resulting from Zika virus infection and the neurodevelopmental, neurological, and urinary track disorders in children. We see a clear lead in research from Brazil and other Latin American countries, given that there was a pandemic in those areas [12] with a percentage of 3720 confirmed CZS cases [41].

The findings, according to the included articles, show that the severe neurodevelopment, neurological, and urinary track outcomes have been shown to be related with severe microcephaly, which is an important anthropometric characteristic of CZS [42]. Severe neurological and neurodevelopment outcomes were a more frequent sign in children born with microcephaly. More specifically, there is a strong correlation between the severity of the outcomes with a lower birth head circumference [26,28,29,30,31,34]. Of course, microcephaly of any type is considered a major driver of neurology and neurodevelopment delays [43]. However, the fact that neurodevelopmental and neurological disorders were observed also in children born asymptomatically indicates that the exposed children without CZS appeared at risk for neurodevelopmental disorders in all of the domain disorders, such as motor, cognition, and mainly in language [20,27,32,35]. Furthermore, normocephalic children should also be under frequent follow-up, due to an increased risk of developing microcephaly after birth [26].

Certainly, the presence of microcephaly and its severity does not only affect the child’s nervous and developmental system. Until today, there are not enough reports on the effects of Zika virus on the urinary tract system and only a few studies and a case report have reported a possible correlation [22,23,24,26,39,40]. Urinary tract disorders are a common condition among children with CZS with severe microcephaly. On a research level, it was observed that the mechanism of Zika virus infection induced acute kidney injury, increasing the levels of the related biomarkers in mice [40]. In addition, some cohorts have reported various disorders some of which are neurogenic bladder, urinary tract infections, bladder agenesis, and abnormal ultrasound findings [22,23,24,26,38], while further research is needed to understand the long-term behavior of Zika virus on the urinary track system. At this point, it should be mentioned that children with CZS are monitored from a very early age by many medical specialties, and it is a bit difficult to progress to kidney failure through neurogenic bladder, as it is a process that is often silent, but takes time to evolve.

The extent of microcephaly at birth is the only significant factor associated with developmental, neurological, and urinary tract infection outcomes so far. Even in normocephalic children, higher rates of developmental delays have been reported. Those children are more likely to appear with a developmental disorder as they get older. However, infants with vertical exposure to Zika virus demanded a longer follow-up to evaluate the development of the disorder and its comorbidities, such as bowel dysfunction and cryptorchidism (34%) [44].

Finally, given the limitations of studies conducted mostly in Brazil’s national rehabilitation centers, more cohort studies need to be conducted on the effects of Zika virus on children from all of the affected countries.

## 5. Conclusions

**Figure 2:**
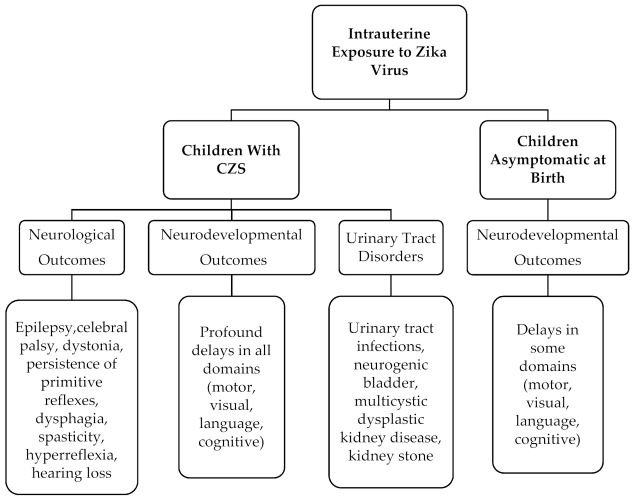
Neurological, neurodevelopmental, and urinary track outcomes in intrauterine exposed to Zika virus children.

An attempt was made in the present systematic review to find out the conditions under which Zika can lead to varying severity of neurodevelopmental, neurological, and urinary tract disorders in children born during the Zika virus outbreak. Although the Zika virus outbreak is no longer a pandemic, it continues to be a major public health problem, due to the high number of congenital malformations as well as the co-occurrence disorders it has caused. In addition, new confirmed cases of Zika virus continue to be reported until today [15], suggesting Zika virus as a continuing threat to maternal and child health. Our study linked CZS and severe microcephaly to the appearance of neurodevelopmental, neurological, and urinary tract disorders. Children with CZS are more likely to have severe neurological, neurodevelopmental, and urinary track effects than children born asymptomatically who are more at risk for neurodevelopmental disorders (Figure 2). Furthermore, Zika virus outcomes have disproportionately affected the poorest families in Latin American countries with significant effects on the economy and society facing the challenges of an uncertain disease. In order to better understand the relationship between the severity of microcephaly at birth and the developmental, neurological, and urinary tract outcomes in early childhood, further research with cohort studies will be needed.

Moreover, ensuring equal access to health services and frequent follow-up, will improve the quality of life of these children, their families, and their health care providers, as well.

## Figures and Tables

**Table 1:** Evaluation of surveying methodological quality.

Author/Year	Selection 1 2 3 4	Comparability 5 6	Result 7 8 9	Total
1.Wheather (2020) [25]	* - * *	* *	* * *	8
2. Pereira (2020) [26]	* - * *	* *	* * *	8
3. Pecanha (2020) [20]	* - * *	* *	* * *	8
4. Mulkey (2020) [27]	* - * *	- *	* * *	7
5. Ferreira (2018) [28]	* - * *	* *	* * *	8
6. Carvalho (2019) [29]	* - * *	- *	* * *	7
7. Bertolli (2020) [30]	* - * *	* *	* * *	8
8. Silva (2020) [31]	* - * *	- *	* * *	7
9. Cranston (2020) [32]	* - * *	- *	* * *	7
10. Garcia-Boyano (2020) [33]	* - * *	* *	* * *	8
11. Quilião (2020) [34]	* - * *	- *	* * *	7
12. Ticona (2021) [35]	* - * *	* *	* * *	8
13. Hcini (2021) [36]	* - * *	- *	* * *	8
14. Abtibol-Bernardino (2020) [37]	* - * *	* *	* * *	8
15. Costa Monteiro (2018) [22]	* - * *	- *	* * *	7
16. Costa Monteiro (2019) [23]	* - * *	- *	* * *	7
17. de Medeiros (2021) [24]	* - * *	* *	* * *	8
18. Villamil-Gómez (2019) [38]	- - * -	- *	* * *	5
19. Liu (2019) [39]	- - **	- *	* * *	6

Notes: 1. Representative exposure sample; 2. selection of non-exposed; 3. exposure finding; 4. outcome did not precede the study; 5. adaptation for mother’s educational level; 6. adaptation for additional confounding factor; 7. outcome evaluation; 8. adequate monitoring time; 9. non-bias of wear. The symbol (*) means that the study met the specific criterion and the symbol (-) means that the study did not meet it.

**Table 2:** Studies included in the review.

Author/ Year	Design	Start– Expiry	N	Population Characteristics	Data/ Country	Outcome
1.Wheather (2020) [25]	longitudinal study	January 2018–Still on going	121	2.5–3 years old CZS	Rehabilitation center Brazil	Profound delays in all neurodevelopmental domains
2. Pereira (2020) [26]	Cohort study	November 2015–2017	75	26–40 months of age Exposed to Zika virus	Instituto de Pesquisa Brazil	Neurological outcomes Urinary tract disorders
3. Pecanha (2020) [20]	Cohort study	May 2016 and January 2018	84	2 years old Asymptomatic at birth	Instituto Fernandes Figueira Brazil	Neurodevelopmental outcomes
4. Mulkey (2020) [27]	Cohort study	1August, 2016– 30 November 2017	70	18 months of age Asymptomatic at birth	National Medical Center Columbia	Neurodevelopmental outcomes
5. Ferreira (2018) [28]	Cross- sectional study	September 2017– January 2018	34	21 months average age CZS	Four rehabilitation facilities Brazil	Neurodevelopmental outcomes
6. Carvalho (2019) [29]	Cohort study	July 2015– 2017	69	23–32 months of age CZS	Neurorehabilitation Hospital Brazil	Neurological outcomes Neurodevelopmental outcomes
7. Bertolli (2020) [30]	Cohort study	July 2017– October 2017	120	19–26 months of age Exposed to Zika virus	The Brazilian Ministry of Health The CDC	Neurological outcomes Neurodevelopmental outcomes
8. Silva (2020) [31]	Cross- sectional study	February 2017– August 2019	219	10–45 months of age Exposed to Zika virus	Two Tertiary Hospitals Brazil	Neurological outcomes Neurodevelopmental outcomes
9. Cranston (2020) [32]	Retrospective cohort study	May 2019– July 2019	219	6–42 months of age Exposed to Zika virus	Instituto Fernandes Figueira Brazil	Neurological outcomes Neurodevelopmental outcomes
10. Garcia-Boyano (2020) [33]	Cohort study	March 2016– September 2019	21	23.6 months of age Exposed to Zika virus	Pediatric Hospital Ecuador	Neurological outcomes Neurodevelopmental outcomes
11. Quilião (2020) [34]	Cohort study	October 2018– February 2020	11	36 months median age CZS	University of Mato Grosso do Sul Brazil	Neurological outcomes Neurodevelopmental outcomes
12. Ticona (2021) [35]	Cohort study	January 2015– December 2016	46	11–32 months of age Exposed to Zika virus	Hospital Brazil	Neurodevelopmental outcomes
13. Hcini (2021) [36]	Cohort study	January 2016– September 2016	129	3 years of age Exposed to Zika virus	Pediatric clinic French Guiana	Neurodevelopmental outcomes Neurological outcomes
14. Abtibol-Bernardino (2020) [37]	Cohort study	2006–2008	26	25–42 months of age Exposed to Zika virus	Tropical Medicine Foundation Brazil	Neurodevelopmental outcomes Neurological outcomes
15. Costa Monteiro (2018) [22]	Cohort study	June 2016– May 2017	22	9.8 months mean age CZS	CZS clinics Brazil	Urinary track Disorders
16. Costa Monteiro (2019) [23]	Cohort study	June 2016–May 2018	69	13.6 months of age CZS	Referral center Brazil	Urinary track Disorders
17. de Medeiros Francilai de Campos (2021) [24]	Cross- sectional study	January 2019– December 2019	33	35–47 months of age CZS	Center for urinary disorders Brazil	Urinary track Disorders
18. Villamil-Gómez (2019) [39]	Case report	February 2017	1	16 weeks of gestation infected fetus	University Hospital Colombia	Urinary track Disorders
19. Liu (2019) [40]	Clinical study	February 2016	-	Newborn and adult mice	Sun Yat-sen University China	Urinary track Disorders

Notes: We defined “exposed children” as those whose mothers were infected by Zika virus during the prenatal period. We defined “infected children” as those whose infection during the perinatal period was confirmed by laboratory and anthropometric tests after birth. We defined “microcephaly” as head circumference smaller than 2 SDs, depending on the sex and age and its anthropometric feature of CZS. As an outcome, we analyzed the neurodevelopmental profound delays in all of the domains (motor, visual, language, cognitive), the neurological (epilepsy, celebral palsy, dystonia, persistence of primitive reflexes, dysphagia, spasticity, hyperreflexia, hearing loss), and the urinary (urinary tract infections, neurogenic bladder, multicystic dysplastic kidney disease, kidney stone) profile in all intrauterines exposed to Zika virus children.

## Data Availability

Not applicable.
